# Abnormal Hippocampal Melatoninergic System: A Potential Link between Absence Epilepsy and Depression-Like Behavior in WAG/Rij Rats?

**DOI:** 10.3390/ijms19071973

**Published:** 2018-07-06

**Authors:** Slavianka Moyanova, Antonio De Fusco, Ines Santolini, Roberta Celli, Domenico Bucci, Federica Mastroiacovo, Giuseppe Battaglia, Ferdinando Nicoletti, Jane Tchekalarova

**Affiliations:** 1 I.R.C.C.S. Neuromed, 86077 Pozzilli, Italy; moyanova.slavianka@neuromed.it (S.M.); antonio.defusco85@gmail.com (A.D.F.); ines.santolini@neuromed.it (I.S.); robertacelli85@hotmail.it (R.C.); domenico.bucci@neuromed.it (D.B.); federica.mast@neuromed.it (F.M.); giuseppe.battaglia@neuromed.it (G.B.); ferdinandonicoletti@hotmail.com (F.N.); 2 Department of Physiology and Pharmacology, Sapienza University of Rome, 00161 Rome, Italy; 3 Institute of Neurobiology, Bulgarian Academy of Sciences, 1113 Sofia, Bulgaria

**Keywords:** absence seizures, pinealectomy, depression, melatonin, MT receptors

## Abstract

Absence epilepsy and depression are comorbid disorders, but the molecular link between the two disorders is unknown. Here, we examined the role of the melatoninergic system in the pathophysiology of spike and wave discharges (SWDs) and depression-like behaviour in the Wistar Albino Glaxo from Rijswijk (WAG/Rij) rat model of absence epilepsy. In WAG/Rij rats, SWD incidence was higher during the dark period of the light-dark cycle, in agreement with previous findings. However, neither pinealectomy nor melatonin administration had any effect on SWD incidence, suggesting that the melatoninergic system was not involved in the pathophysiology of absence-like seizures. Endogenous melatonin levels were lower in the hippocampus of WAG/Rij rats as compared to non-epileptic control rats, and this was associated with higher levels of melatonin receptors in the hippocampus, but not in the thalamus. In line with the reduced melatonin levels, cell density was lower in the hippocampus of WAG/Rij rats and was further reduced by pinealectomy. As expected, WAG/Rij rats showed an increased depression-like behaviour in the sucrose preference and forced swim tests, as compared to non-epileptic controls. Pinealectomy abolished the difference between the two strains of rats by enhancing depression-like behaviour in non-epileptic controls. Melatonin replacement displayed a significant antidepressant-like effect in both WAG/Rij and control rats. These findings suggest that a defect of hippocampal melatoninergic system may be one of the mechanisms underlying the depression-like phenotype in WAG/Rij rats and that activation of melatonin receptors might represent a valuable strategy in the treatment of depression associated with absence epilepsy.

## 1. Introduction

The hormone melatonin possesses neuromodulatory properties, exerts inhibitory function in the central nervous system (CNS) and regulates the circadian rhythms. The main source of melatonin in the brain is the pineal gland. Exogenously delivered melatonin has antioxidant [1], neuroprotective [2], anticonvulsant [3], as well as anxiolytic effects [4,5] in rodents. Long-term treatment with this hormone corrects disease-induced neuronal damage and behavioral disturbances in a variety of models [6,7,8]. Recently, we have found that melatonin treatment before or after kainate (KA)-induced status epilepticus (SE) exerts a strain-dependent effect on oxidative stress [9] and development of epileptogenesis [10,11]. Melatonin treatment suppresses KA-induced seizures only during the period of treatment in Wistar rats [10] and causes a long-lasting reduction of motor seizures in spontaneously hypertensive rats [11].

Previous studies have shown that the removal of the pineal gland, which causes a substantial decrease in the plasma melatonin levels, increases the seizure threshold, thereby facilitates the development of epileptogenic processes in different seizure models [12,13,14]. Pinealectomy shortens the latent seizure-free period in the pilocarpine model of temporal lobe epilepsy [12], reduces the number of electrical stimulations of amygdala required for kindling development [13], and decreases the latency of initial epileptiform discharges in the penicillin model of focal epilepsy [14]. In addition, pinealectomy exacerbates pilocarpine-induced neuronal death and mossy fiber sprouting in the hippocampus [12]. Melatonin replacement reverses the pro-convulsant effect of pinealectomy in the pilocarpine model of SE and penicillin model of focal epilepsy [12,14]. Melatonin has shown to exert both pro- and anticonvulsant effects in humans with intractable seizures [15,16]. There are only a few data on the effect of melatonin on absence epilepsy. These data are limited to two articles in which melatonin influences spike-wave discharges (SWDs) in the Wistar Albino Glaxo from Rijswijk (WAG/Rij) rat model of absence epilepsy [17,18]. WAG/Rij rats develop SWDs associated with behavioral arrest after 3–4 months of age, and represent a valuable model of absence epilepsy in humans with lack of responsiveness during SWDs, their increased incidence at transitions among the sleep phases, inheritance and effectiveness of anti-absence drug ethosuximide but not that of other anti-convulsant drugs such as tiagabine, carbamazepine and diphenylhydantoin against SWDs [19,20]. The WAG/Rij strain does not recapitulate, however, two other aspects of absence epilepsy, the late appearance of the SWDs during life span in rats compared to early manifestation of absence seizures in childhood of humans; and the frequency difference of the spikes and waves in the SWD episodes [20]. Interestingly WAG/Rij rats display a depression-like phenotype [21], and therefore can be also used for study of mechanisms underlying the established comorbidity between absence epilepsy and depression in absence epilepsy [22,23]. This gave us impetus to study the effects of pinealectomy and melatonin replacement in WAG/Rij rats because the suprachiasmatic/pineal gland/melatonin axis is critically involved in the pathophysiology of mood disorders and sleep abnormalities associated with these disorders.

We now report that the manipulation of the melatonin axis has no impact on the incidence and the circadian periodicity of absence seizures in WAG/Rij rats, but has a profound effect on depression-like behavior in these animals. In addition, we show that the hippocampal melatoninergic system is abnormal in WAG/Rij rats, and this may provide a potential link between absence seizures and comorbid depression-like phenotype.

## 2. Results

### 2.1. Melatonin Had No Role in the Circadian Rhythm of Absence Seizures and No Impact on their Incidence in WAG/Rij Rats

**Figure 1:**
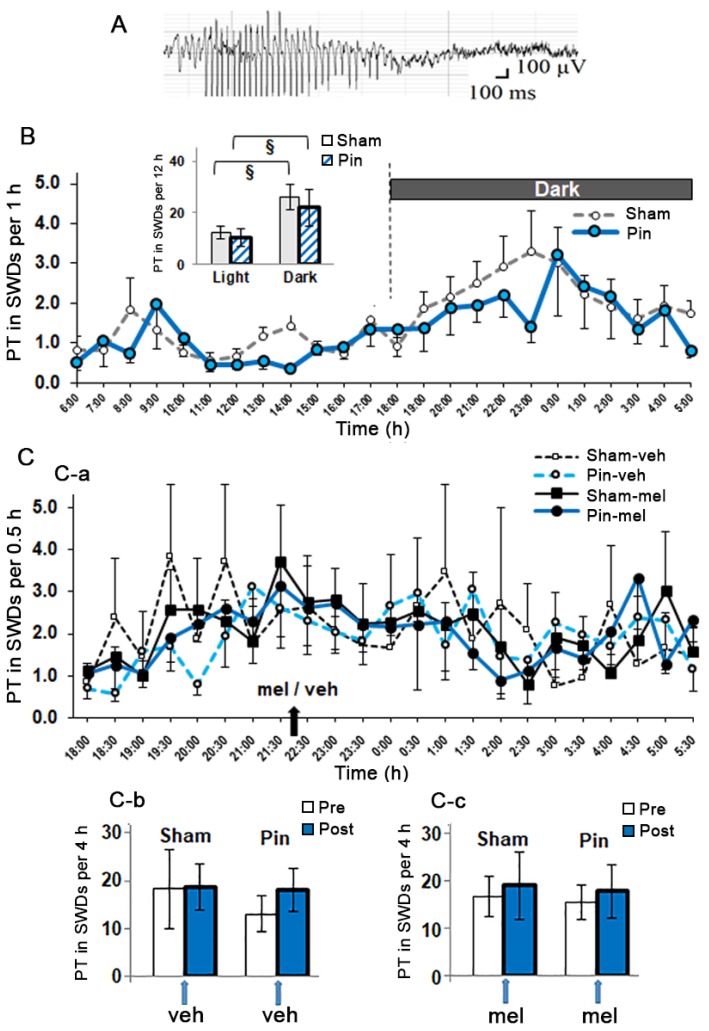
Effects of pinealectomy and melatonin treatment on SWDs in Wistar Albino Glaxo from Rijswijk (WAG/Rij) rats. Representative SWD and PT in SWDs per hour of recording from 6:00 a.m. (light on) to 6:00 p.m. (light off) in Sham and pinealectomized (Pin) WAG/Rij rats are shown in (**A**,**B**), respectively. Total PT in SWDs during the entire light and dark phases is shown in the inset. (§)—Significant difference between light and dark phases for both Pin and Sham rats [2-way ANOVA and post-hoc Fisher least-significant difference (LSD) test, *p* < 0.05]. The effects of treatment with melatonin (80 mg·kg^−1^ s.c.) and vehicle in Sham and Pin rats on PT in SWDs are shown in (**C**). Injections were performed at 10:00 p.m. (arrow in (**C-a**)). SWDs were counted from the second 30-min period after injections to minimize the effect of stress on the incidence of SWDs. Total PTs in SWDs in the 4 h preceding and following vehicle or melatonin treatments in Sham and Pin rats are shown in (**C-b**) and (**C-c**), respectively. Values are means ± S.E.M.

The electrographic profile of SWDs and the dynamics of the hourly percent time (PT, time spent in SWDs in percent) over 24 h in WAG/Rij rats are shown in Figure 1A,B.

Two-way analysis of variance with repeated measures (rANOVA) showed significant effect of light-dark Phase [F_(1,5)_ = 15.51, *p* = 0.011], as well as Phase x Hour interaction [F_(11,55)_ = 2.27, *p* = 0.023] on PT in SWDs for the 12-h duration of light and dark phases in intact WAG/Rij rats. The acrophase of SWD incidence was between 11:00 p.m. and 0:00 a.m. Total PT in SWDs during dark was approximately two-fold higher than during light: 26.08 ± 4.85% and 12.53 ± 2.37%, respectively (Figure 1B, inset). Pinealectomy did not affect the PT in SWDs both during the dark and light phases: [F_(1,8)_ = 0.28, *p* = 0.61]. Melatonin administered just before the acrophase (at 10:00 p.m.) did not affect PT in SWDs neither in pinealectomized WAG/Rij rats nor in rats without pinealectomy (Sham) (Figure 1C-a). Three-way repeated measure analysis of variance (rANOVA) did not demonstrate main effects of Group (Sham, Pin), Drug (mel, veh) and Time (each 0.5 h epoch). Post-hoc comparisons did not reveal any significant difference between total PTs in SWDs in 4 h before and 4 h after melatonin injection, neither between Sham and Pin rats, nor between vehicle and melatonin injections in both groups (Figure 1C-b,C-c). Taken together these data suggest that melatonin has no major role in the increased PT in SWDs observed during the dark phase in WAG/Rij rats.

### 2.2. Abnormalities in Hippocampal Melatonergic System in WAG/Rij Rats

**Figure 2:**
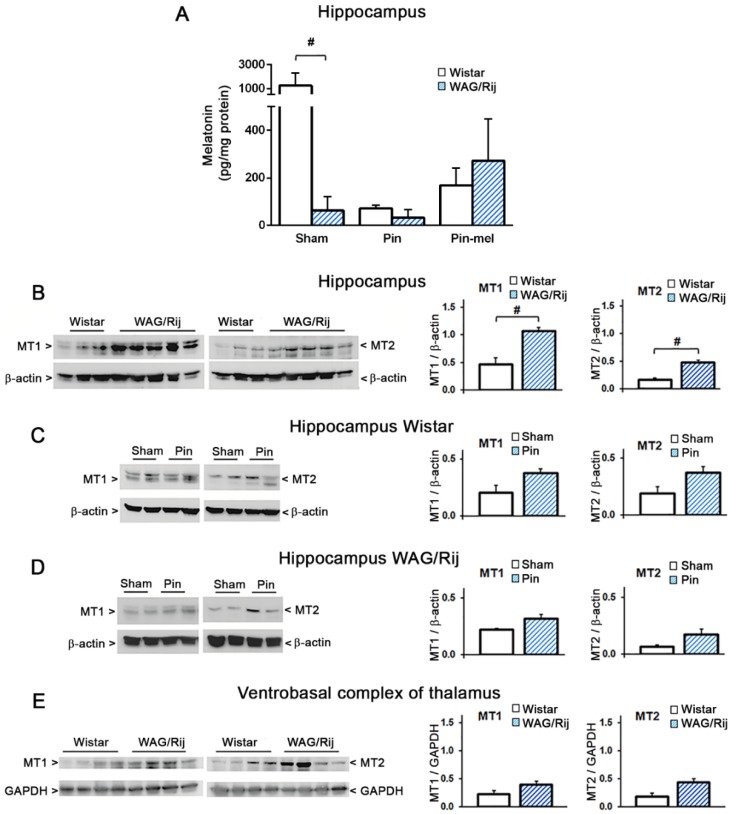
Changes in hippocampal melatonin and levels of MT1 and MT2 protein receptor levels in WAG/Rij rats. (**A**) Hippocampal levels of melatonin in Wistar and WAG/Rij rats under basal conditions (Sham), 24 days after surgical removal of the pineal gland (Pin), and after melatonin replacement (10 mg/kg, s.c., daily for 18 days, administered at 5.30 p.m.). (#) *p* < 0.05 between intact WAG/Rij and Wistar rats; (**B**) Western blot analysis of MT1 and MT2 receptors in the hippocampus of Wistar and WAG/Rij rats. (#) *p* < 0.05 between WAG/Rij and Wistar rats; (**C**) Effect of pinealectomy on hippocampal MT1 and MT2 receptor levels in Wistar rats and the representative Western blots on the left; (**D**) Effect of pinealectomy on hippocampal MT1 and MT2 receptor levels in WAG/Rij rats and the representative Western blots on the left; (**E**) Western blot analysis of MT1 and MT2 receptor protein levels in the ventrobasal thalamus of Wistar and WAG/Rij rats. All values are means + S.E.M. Statistical analysis was performed using the Mann–Whitney U test.

Endogenous melatonin levels were largely reduced in the hippocampus of WAG/Rij rats, as compared to age-matched non-epileptic Wistar rats. Melatonin levels in intact WAG/Rij rats were as lower as levels observed in pinealectomized Wistar rats (95%↓) (Figure 2A). The estimated effect size [24] (coefficient Cohen’s d = 0.94, percent overlap (OL) = 46%) showed that 54% of WAG/Rij rats were reliably sensitive to the reduction of the melatonin level in hippocampus versus 46% of Wistar rats. Pinealectomy largely reduced melatonin levels in Wistar rats but had only small effects in WAG/Rij rats. Subcutaneous melatonin replacement after pinealectomy (10 mg/kg, once a day for 18 days) raised hippocampal melatonin levels in both strains of rats, but not to the extent found in the intact Wistar rats.

We also compared the expressions of the MT receptors in the hippocampus of Wistar and WAG/Rij rats. As compared to Wistar rats, WAG/Rij rats showed higher MT1 (128%↑, Cohen’s d = 3.4, OL = 5%) and MT2 (182%↑, Cohen’s d = 3.8, OL = 3%) receptor protein levels in the hippocampus (Figure 2B). There was a trend for pinealectomy to increase the hippocampal levels of MT1 and MT2 receptor protein levels in both strains of rats, Wistar and WAG/Rij (*p* values between 0.05 and 0.1). The additional analysis of the effect sizes (Cohen’s d coefficients and percent OL, [24]) showed, however, that the increase in the hippocampal MT1 receptor levels with 45%↑ (Cohen’s d = 2.2, OL = 16%) obtained in 84% of pinealectomized WAG/Rij rats, and the increase in the MT2 receptor levels with 143%↑ (d = 1.8, OL = 23%) obtained in 77% of the same WAG/Rij rats differed substantially from the corresponding values obtained in sham rats (16% and 23%). We extended the analysis to the ventrobasal thalamus, which is a key region in the cortico-thalamic-cortical network involved in SWDs [25]. We did not find significant difference between Wistar and WAG/Rij rats for both MT1 and MT2 receptor expressions in the ventrobasal complex of thalamus (Figure 2E).

### 2.3. Reduced Hippocampal Cell Density in WAG/Rij Rats

**Figure 3:**
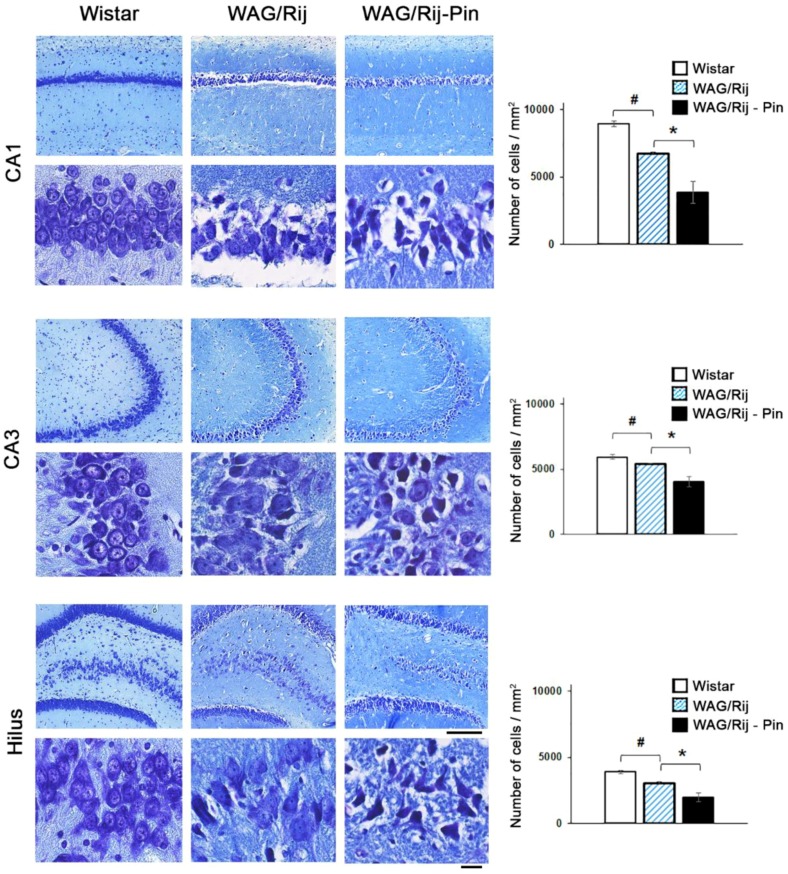
Cell density in the hippocampus of Wistar and WAG/Rij rats, and effects of pinealectomy in WAG/Rij rats. Representative Nissl-stained sections of CA1, CA3 and Hilus are shown on the left. Scale bars: 200 µm for 10× and 20 µm for 60×. Values of cell densities are means ± S.E.M. for Wistar-Naive; WAG/Rij-Sham, and WAG/Rij-Pin. *p* < 0.05 between intact WAG/Rij and Wistar rats (#), or between WAG/Rij-Pin versus WAG/Rij-Sham rats (*). Statistical analysis was performed with the Mann–Whitney U test.

Knowing that the lack of melatonin causes cell death in the hippocampus [26,27], we counted cell number after Nissl staining in WAG/Rij and non-epileptic Wistar rats. Interestingly, cell density was significantly reduced in three hippocampal subregions (CA1, CA3, and Hilus) of WAG/Rij rats compared to that in Wistar rats (Figure 3), as follows: CA1 (25%↓, d = 6.4, OL = 2%); CA3 (9%↓, d = 2.3, OL = 14%); Hilus (22%↓, d = 3.9, OL = 2.6%). The coefficients Cohen’s d and percent overlaps (OL) calculated according to [24] showed that more than 86% of WAG/Rij rats were reliably sensitive to the cell loss in these three hippocampal fields versus only 14% of Wistar rats. Pinealectomy further decreased hippocampal cell density in WAG/Rij rats (Figure 3): CA1 (45%↓, d = 2.4, OL = 13%); CA3 (25%↓, d = 2.0, OL = 19%); and Hilus (36%↓, d = 1.8, OL = 23%), which means that more than 77% of Pin rats had cell density loss in hippocampus not obtained in Sham rats, and these effects might be interpreted as large [24].

### 2.4. Influence of Melatonin on Depression-Like Behavior in WAG/Rij and Non-Epileptic Wistar Rats

**Figure 4:**
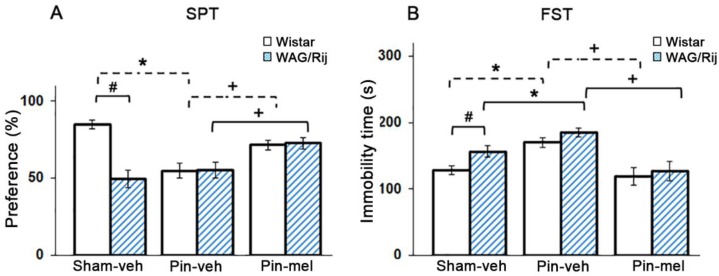
Depression-like behavior in Wistar and WAG/Rij rats. Effects of strain (WAG/Rij and Wistar rats), pinealectomy (Sham and Pin rats) and drug (sub-chronic 18-day-treatment with melatonin, 10 mg·kg^−1^ s.c. or its vehicle) on saccharine preference in the SPT and immobility time in the FST are shown in (**A**,**B**), respectively. (**#**) *p* < 0.05 (Student’s *t* test) between Wistar and WAG/Rij rats; (*) *p* < 0.05 between Sham and Pin rats, treated with vehicle (two-way ANOVA (Strain and Pinealectomy) and Fisher LSD test); (**+**) *p* < 0.05 between vehicle-treated and melatonin-treated Pin rats (two-way ANOVA (Strain and Drug) and Fisher LSD test). Values are means ± S.E.M.

For the analysis of depression-like behavior, we used two behavioral tests: (i) the saccharine preference test (SPT) for assessment of anhedonia; and (ii) the forced swim test (FST) as a despair-like behavior. As expected, WAG/Rij rats showed an increase in depressive-like behavior (less preference to saccharine solution in SPT (41%↓, d = 1.8, OL = 23%) and greater immobility time in FST as compared to Wistar rats (22%↑, d = 1.0, OL = 45%) (Figure 4). The d coefficients of Cohen and percent OLs showed that more than 77% of WAG/Rij rats had decreased preference in SPT and more than 55% of them had increased immobility time not obtained by Wistar rats.

Pinealectomy abolished the difference between Wistar and WAG/Rij rats in both behavioral tests by increasing the depression-like behavior in Wistar rats (less preference 35%↓, d = 2.5, OL = 12% in SPT and 33%↑, d = 1.7, OL = 25% in FST in Wistar rats with pinealectomy vs. sham Wistar rats). Pinealectomy did not change sucrose preference but slightly increase the immobility time (18%↑, d = 0.9, OL = 48.4%) in WAG/Rij rats. Two-way ANOVA revealed a significant effect of Strain [F_(1,48)_ = 8.19, *p* = 0.006] and pinealectomy [F_(1,48)_ = 4.09, *p* = 0.04] (Figure 4A). Melatonin replacement after pinealectomy for 18 days caused a substantial antidepressant-like effect in the SPT in both Wistar (31%↑, d = 1.5, OL = 29%) and WAG/Rij (31%↑, d = 1.2, OL = 38%) rats [F_(1,33)_ = 10.19, *p* = 0.003] (Figure 4A). Sub-chronic treatment with melatonin produced significant antidepressant effects also in the FST for both Wistar (30%↓, d = 1.6, OL = 27%) and WAG/Rij rats (31%↓, d = 1.6, OL = 27%) [two-way ANOVA: F_(1,45)_ = 29.12, *p* < 0.000001] (Figure 4B). The d coefficients of Cohen and percent of OL showed that the effects of melatonin substitution might be classified as large according the Cohen’s standard (see [24]).

## 3. Discussion

Clinical studies indicate that epilepsy and depression are comorbid disorders in both children and adults, and comorbidity is also found in patients affected by genetic generalized epilepsies, such as juvenile myoclonic epilepsy and absence epilepsy [28,29,30,31,32]. The cause-to-effect relationship between depression and absence or convulsive epilepsy is uncertain, and whether there is a common neurobiological substrate for both phenotypes or, rather, psychosocial or iatrogenic factors account for the comorbidity, remains to be determined. WAG/Rij rats, which develop age-dependent, spontaneous absence seizures, have been described as a genetic animal model of absence epilepsy and mild comorbid depression [33]. These animals show decreased investigative activity in the open field, increased immobility in the FST, and reduced sucrose preference in the SPT [33,34], also found in the present study. Using the WAG/Rij model, Russo and co-workers [34] have demonstrated that the atypical antipsychotic aripiprazole, improves depression-like behavior and cognitive functions associated with absence epilepsy, a finding of potential translational value for drug treatment in humans. Understanding why WAG/Rij rats exhibit a mild depression-like phenotype might shed new light into the biological link between absence seizures and mood disorders. 

In agreement with previous findings [35], we found here that SWDs in WAG/Rij rats were more frequent during dark (see Figure 1). We did not examine whether and how the rest activity-sleep state influences frequency of seizures appearance in WAG/Rij rats, but it is known that the inactive state in nocturnal rodents positively associates with absence seizures, with SWDs being more numerous during passive waking, during the first stage of the slow-wave sleep, and at transitions among the sleep phases [19]. In contrast, convulsive seizures show an opposite circadian rhythmicity, with limbic seizures being more frequent during light in both rats and humans [36,37]. Although the exact mechanism responsible for the periodicity of absence seizures remains unknown, it was reasonable to hypothesize an involvement of the suprachiasmatic nucleus/pineal gland axis because melatonin is secreted during dark in all animal species [38]. However, at least in our study, neither pinealectomy nor melatonin replacement during dark had any effect on SWDs in WAG/Rij rats. The lack of effects of pinealectomy and melatonin administration on SWDs in our study was unexpected because it has been reported that melatonin reduces the incidence of SWDs in intact (non-pinealectomized) WAG/Rij rats [17,18]. The reasons for these contrasting findings are unknown. At least in our WAG/Rij rats, melatonin does not seem to have a role in the generation, incidence, and circadian pattern of absence seizures. In addition, expression of MT1 and MT2 melatonin receptors in the thalamus did not differ between WAG/Rij rats and age-matched non-epileptic controls.

We were surprised to find that hippocampal melatonin levels in WAG/Rij rats were reduced to an extent similar to that observed in pinealectomized Wistar rats, and, perhaps as a consequence of this, MT1 and MT2 receptors were up-regulated in the hippocampus of WAG/Rij rats. We could not measure blood melatonin in WAG/Rij rats, and, therefore, we do not know whether the decrease in hippocampal melatonin levels was due to a reduced activity of the pineal gland, to an accelerated melatonin clearance, or to a reduced local production of melatonin in the hippocampus. An extrapineal production of melatonin has been demonstrated in many organs, including the brain, and melatonin-synthesizing enzymes are present in different brain structures [39]. However, the large reduction in hippocampal melatonin levels after pinealectomy in non-epileptic Wistar rats suggested that the pineal gland was a major, if not unique, source for hippocampal melatonin. It will be interesting to examine whether changes in melatonin levels are also present (or not) in other brain regions of WAG/Rij rats.

We and others have found that pinealectomy causes cell death in the hippocampus, and cells are rescued by melatonin replacement [26,27]. In line with the large reduction of local melatonin levels, WAG/Rij rats showed a reduced cell density in the hippocampus. This supports the view that neurons in the dorsal hippocampus are highly sensitive to the pro-survival and neurotrophic activity of melatonin [10]. In WAG/Rij rats, pinealectomy further decreased hippocampal cell density, although it caused only a small trend to a reduction in melatonin levels. One possible explanation is that there were additional mechanisms by which pinealectomy caused hippocampal cell death. For example, pinealectomy disrupts the physiological balance between melatonin and the hypothalamic-pituitary-adrenal (HPA) axis, resulting in an uncontrolled secretion and activity of glucocorticoids (corticosterone in rats) [27,40,41], which, in turn, might contribute to hippocampal cell death [42,43]. This interesting hypothesis warrants further investigation.

It was logical to examine whether abnormalities in hippocampal melatoninergic system could be related to the depression-like phenotype of WAG/Rij rats. In agreement with previous findings [33], our WAG/Rij rats showed a reduced sucrose preference in the saccharine preference test and an increased immobility time in the forced swim test. Both tests have pharmacological validity and are used for the screening of antidepressant medication (reviewed by [33]). The large difference in hippocampal melatonin levels between WAG/Rij and non-epileptic control rats precluded any comparison between the two strains of rats in response to a fixed dose of exogenous melatonin. Thus, we decided to circumvent the obstacle by examining the effect of pinealectomy on depression-like behavior in epileptic and non-epileptic rats. Interestingly, pinealectomy abolished any difference between WAG/Rij and Wistar rats. This was due to a pro-depressant effect of pinealectomy in non-epileptic Wistar rats. In WAG/Rij rats, pinealectomy did not further reduce sucrose preference and only slightly enhanced the immobility time in the FST. The lack of significant behavioral effects of pinealectomy in WAG/Rij rats was expected because these rats already showed very low melatonin levels in the hippocampus. Melatonin treatment caused an antidepressant-like effect in both WAG/Rij and Wistar rats after pinealectomy, i.e., in a condition in which hippocampal melatonin levels were similar in both strain of rats.

Abnormalities of serotonergic and dopaminergic transmission are central in the pathophysiology of depressive disorders [31,44,45]. The despair-like state in the FST is associated with abnormalities in the reciprocal connections between dorsal raphe nucleus (the major source of forebrain serotonin) and the prefrontal cortex, whereas anhedonia in the SPT reflects a defective activity in the mesolimbic dopaminergic system projecting from the ventral tegmental area to the nucleus accumbens [46]. Alterations in serotonergic and dopaminergic neurotransmission have also been related to comorbidity between absence seizures and depression in WAG/Rij rats [33]. A defective melatoninergic system might contribute to these changes because melatonin modulates serotonin synthesis and the activity of serotonergic neurons in the brain [47,48,49]. 

In conclusion, our findings demonstrate that, at least in the WAG/Rij model of absence epilepsy, melatonin is not involved in the incidence or circadian pattern of SWDs, but, rather, may link the epileptic phenotype to comorbid depression. A defective hippocampal melatoninergic system may well account for the depression-like behavioral phenotype of WAG/Rij rats. Whether this defect extends to other brain regions that are critically involved in the pathophysiology of mood disorders remains to be determined. An important question is whether the reduction in hippocampal melatonin levels in WAG/Rij rats is secondary to SWDs, or, rather, this is a primary defect that could be genetically determined. Studies with antiabsence drugs are needed to clarify this issue. Our data lay the groundwork for further preclinical studies aimed at establishing whether treatment with melatonin or other agonists of MT receptors (e.g., the antidepressant agomelatine) is particularly effective in the management of depression associated with absence epilepsy.

## 4. Materials and Methods

The experimental protocol was approved by the Ethical Committee of Neuromed I.R.C.C.S. (Pozzilli, Italy) and by the Italian Ministry of Health (n. 1088/2015-PR, 19 Oct. 2015).

### 4.1. Animals

**Scheme 1:**
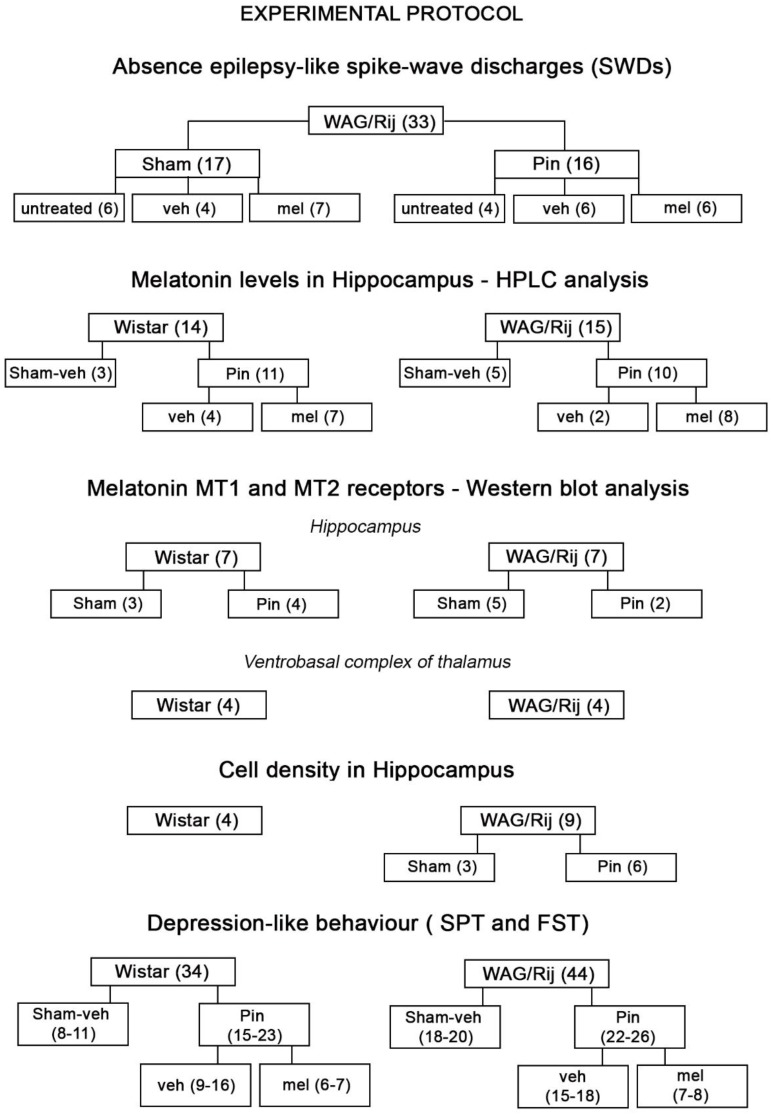
Schematic illustration of the experimental protocol. Groups and number of animals used are shown. The number of animals for the behavioral experiments are shown as total number per group for both tests (SPT and FST). The animals used for determination of melatonin levels and expressions of melatonin receptors were from the cohort of animals used for behavioral experiments. The WAG/Rij rats used for determination of cell loss in hippocampus were from the cohort of animals used for electrophysiological experiments.

The experiments were performed on male inbred WAG/Rij rats (five/six-month old) and age-matched Wistar rats housed in the animal facility of I.R.C.C.S. Neuromed Institute (Pozzilli, Italy). The animals were housed (3–4 per cage) under standardized conditions (20 ± 3 °C, 40–50% humidity; 12/12-h light/dark cycle, with light on at 6:00 a.m.) and habituated for a week before experiments. Food and water were available ad libitum throughout the study except during test procedures. The experimental protocol with number of animals participating in each experiment is shown in Scheme 1.

### 4.2. Drugs

Melatonin (Sigma-Aldrich, St. Louis, MO, USA), dissolved in peanut oil (Sigma-Aldrich), was injected subcutaneously (s.c.) at a dose of 80 mg·kg^−1^ body weight during the dark phase, at 10:00 p.m. (acute experiment) and at a dose of 10 mg·kg^−1^ for 18 days (subchronic experiment) just before dark, at 5:30 p.m. These doses have previously been shown to be effective in different seizure tests [10,50]. The matched groups received vehicle in the same conditions.

### 4.3. Pinealectomy

Pinealectomy (Pin) was performed following the method described by Hoffmann and Reiter [51] and used in our previous work [26]. Briefly, rats were anesthetized with isoflurane and fixed in a stereotaxic apparatus. A piece of skull was removed at the juncture of lambda and the sagittal suture lines. Then the pineal gland was grasped with fine forceps and removed. In sham-operated rats (Sham), the same procedure was used except that the pineal gland was not removed.

### 4.4. Implantation of Electrodes and Recording and Analysis of SWDs

The implantation of the electrodes was made four-five weeks after the pinealectomy. Two pieces of teflon (PFA)-coated stainless-steel wires (A-M Systems, Sequim, WA, USA) with diameter of 127.0 (bare)/203.2 (coated) µm were inserted into the frontal cortex through small holes in the skull made over homologous points of both hemispheres: 0.5 mm anterioposterior to Bregma, 2.5 mm mediolateral and 1.0–1.4 mm dorsoventral below the dura mater. The wires were cut at the desired length immediately before the insertion without removing the PFA coating at the end and thus the contact area for the electrosubcorticogram (ESCoG) recording was the area of the cross section of the bare wire (area approximately 0.01 mm^2^). For simplicity, the ESCoG will be referred in the paper as electroencephalogram (EEG). Two stainless steel screws (0–80 × 3/32, Plastics One) fixed above the frontal sinus served as indifferent and ground electrodes, and also for fixing the implant to the skull. The electrodes were connected to a miniature 6-way male socket (RS components) and the whole assembly was fixed to the skull with Glass ionomer cement (KetacTM Cem radiopaque). The EEG recordings began at least one week after the electrode implantation and were carried out 24 h (WAG/Rij non-treated rats) or 12 h during the dark phase (WAG/Rij rats, treated with melatonin or its vehicle). EEG was recorded by means of Grass-Technologies 800Hz amplifier and Grass-Telefactor software (Astro-Med, Inc., West Warwick, RI, USA.). The beginning and the end of each SWD episode in the EEG were determined using classical criteria [19] by means of Acknowledge 4.1.1 software (BIOPAC Systems, Inc., Goleta, CA, USA) with a precision of 10 ms. Then, number and mean duration of SWDs in each EEG epoch were calculated in Excel of Microsoft Office (2016). Simultaneous video-recording of behavior was performed as additional criteria for the SWD identification [19]. The PT in SWDs was determined in percent of the time occupied by SWDs in each epoch of EEG (1 h for the 24 h-recordings in non-treated rats and 0.5 h for the 12 h-recordings during dark in rats treated with melatonin/vehicle). The absolute value of PT in SWD was equal to the total number of SWDs in a given period of EEG recording multiplied by the seconds of their mean duration in the same period. The PT in SWD was evaluated in dynamics for each EEG epoch (1 h or 0.5 h) or as a total value for the whole EEG period of interest (12 h of light/dark phases or 4 h before/4 h after acute treatment with melatonin or its vehicle). 

### 4.5. Measurements of Melatonin

Hippocampal melatonin levels were quantitated after an extraction procedure with chloroform. Hippocampi were dissected out and homogenized in RIPA buffer. An aliquot was used for protein determination. Five hundred μL of homogenate were centrifuged in a microfuge at maximal speed for 10 and the supernatant was mixed with 2.5 mL of chloroform. The mixture was vortexed, centrifuged (3000 rpm, 10 min), and the aqueous phase aspirated. The organic phase was separated and cleaned once with 500 μL 0.1 N NaOH. After stirring and centrifugation (3000 rpm, 10 min), the aqueous phase was aspirated and the organic layer was evaporated to dryness overnight at room temperature. The residue was dissolved in 100 μL of mobile phase and centrifuged in a microfuge at maximal speed for 15 min. Twenty μL of the supernatant were injected into the HPLC system.

The HPLC apparatus consisted of a programmable solvent module 126 (Beckman Instrument, Fullerton, CA, USA), an analytical C-18 reverse phase column (Ultrasphere ODS 3 μm Spherical, 80 Å pore, 4.6 mm × 75 mm, Beckman Instrument) and a RF-551 spectrofluorometric detector (Shimadzu, Kyoto, Japan). Excitation and emission wavelengths were set at 285 and 360 nm, respectively. The mobile phase consisted of 85 mM acetic acetate, 0.1 mM EDTA-Na2, and acetonitrile 14% of final volume, pH adjusted to 4.7. All analyses were performed at room temperature at an isocratic flow rate of 1.0 mL/min. Acquisition and integration of chromatograms was performed using the Gold Nouveau software (Beckman Instrument). The identity of melatonin peak was confirmed by spiking with an authentic standard solution of melatonin.

### 4.6. Western Blotting of Melatonin Receptors

The expression of melatonin receptors MT1 and MT2 was estimated by Western blotting, using highly specific polyclonal antibodies (1:2000, for MT1, or 1:500, for MT2, Alomone, Jerusalem, Israel) detect bands of approximately 40 kDa, at expected molecular weight. Hippocampus and ventrobasal complex of thalamus from Wistar and WAG/Rij rats were homogenized at 4 °C in 50 mM Tris-HCl buffer, pH 7.4, containing 1 mM EDTA, 1% Triton X-100, 1 mM PMSF, 1 µg/mL aprotinin, 1 µg/mL pepstatin, and 1 µg/mL leupeptin. After sonication, 2 µL of total extracts were used for protein determinations. One hundred µg of protein extract were resuspended in sodium dodecyl sulfate (SDS)-bromophenol blue reducing buffer with 40 mM dithiothreitol (DTT). Western blot analyses were carried out by loading 35 µg of total proteins per lane into 10% SDS polyacrylamide gels, which were electroblotted on immunoblot polyvinylidene difluoride (PVDF) membranes (BioRad, Milano, Italy). The PVDF membranes were blocked in TBS-T buffer containing 0.25% non-fat dry milk, by using the SNAP i.d. protein detection system (Millipore, Burlington, MA, USA), and incubated with polyclonal MT1 or MT2 antibodies; blots were then incubated with secondary antibodies (peroxidase-coupled anti-rabbit; 1:2000; Calbiochem, San Diego, CA, USA). Immunostaining was revealed by enhanced chemiluminescence luminosity (GE Healthcare, Milan, Italy). The blots were reprobed with a mouse monoclonal antibody to label β-actin (1:100.000, Sigma, St. Louis, MO, USA) or a rabbit polyclonal GAPDH antibody (1:2.500, Abcam, Cambridge, UK) followed by an anti-mouse secondary antibody or an anti-rabbit secondary antibody (1:7000; Calbiochem, San Diego, CA, USA), respectively. Immunoreactive protein bands were quantified using the densitometric method (Scion image software, http://rsb.info.nih.gov/nihimage/). Values were obtained by calculating the ratio between the area under the curve (AUC) of the optical density of MT1 or MT2 signal and the AUC of the house keeping protein for each lane.

### 4.7. Histology

Three months after pinealectomy the animals were deeply anesthetized with isoflurane and sacrificed. Brains were fixed in Carnoy’s solution and embedded in paraffin. Portion of brain included between 1.3 and 6.0 mm posterior from Bregma was sectioned at 10 μm. Sections regularly spaced every 350 μm were deparaffinized and processed for staining with thionin (Nissl staining for histological assessment of neuronal degeneration). Cell density values (number of cells/mm^2^) were calculated. Cells were counted within a square known as disector (40 × 40) μm for CA1, CA3, and hilus region of hippocampus. Disectors were randomly placed by a software (Image Pro Plus 6.2) within areas of interest, drawn by the operator. The operator detected and counted all cells within each disector. The results were expressed as cell density/mm^2^.

### 4.8. Behavioral Tests

#### 4.8.1. Saccharine Preference Test (SPT)

The test was performed as described previously [27]. The rats were placed in individual cages and were supplied with two 100-mL graduated bottles; one filled with water and the other with 1% saccharine. The test was conducted during the dark phase. Saccharine preference was expressed as a percentage of the volume of saccharine solution of the total volume of fluid (saccharine plus regular water) consumed during 12-h period.

#### 4.8.2. Forced Swim Test (FST)

A modified FST was performed according to Mazarati et al. [52]. The test was carried out in a clear and a transparent cylinder (50 cm tall, 25 cm diameter) to a level of 30 cm from the bottom with 24 °C tap water. A single 5-min swimming session was conducted and video-recorded for off-line analysis. The parameter measured was time of immobility (in seconds) when the rat remained motionless or made only movements necessary to keep its head above the water.

### 4.9. Statistical Analysis

Prior to statistical analyses, all data were inspected for outliers and normality to ensure their appropriateness for parametric statistical tests. Both parametric and nonparametric tests were used, depending on variables analyzed. Statistica 7.0 (Statsoft, Inc., Tulsa, OK, USA) statistical package was used for the analysis of all data. The EEG data were analyzed using parametric statistical evaluation with two- or three-way repeated measures rANOVAs. If there were statistical effects of factors Pinealectomy (Sham, Pin), Phase (light, dark), Drug (vehicle, melatonin) and Time (each EEG epoch), post-hoc analysis with Fisher LSD. The behavioral data were analyzed by 2-way ANOVA with factors: Strain (Wistar and WAG/Rij) and Pinealectomy (Sham, Pin), and a second 2-way ANOVA with factors Strain (Wistar and WAG/Rij) and Drug (Vehicle and Melatonin), followed by Fisher LSD test. The difference between WAG/Rij and Wistar rats (Sham, vehicle-treated) was assessed using Student’s t-test. The histological, the HPLC, and Western blot densitometric data were analyzed with nonparametric Mann-Whitney U test. The significant level was set at 0.05. Additionally, percent changes were calculated and effect sizes estimated by means of the Cohen’s d coefficients (Site of the Social Science Statistics assessed on 24 June 2018: http://www.socscistatistics.com/effectsize/Default3.aspx.) The percent overlaps were determined and interpreted according to [24,53].

## Abbreviations

ANOVA	Analysis of variance
AUC	Area under the curve
CNS	Central nervous system
EEG	Electroencephalogram
ESCoG	Electrosubcorticogram
FST	Forced swim test
HPA	Hypothalamic-pituitary-adrenal axis
HPLC	High performance liquid chromatography
KA	Kainic acid (kainate)
LSD	Least significant difference
Mel	Melatonin
MT	Melatonin receptor
Pin	Pinealectomy (rats with removed pineal gland)
PT in SWDs	Percent time in SWDs
rANOVA	Repeated measure analysis of variance
OL	Percent overlap
s.c.	Subcutaneous
SE	Status epilepticus
S.E.M.	Standard error of mean
Sham	Sham-operated (rats without removed pineal gland)
SPT	Saccharine preference test
SWD	Spike-wave discharge
WAG/Rij	Wistar Albino Glaxo from Rijswijk rats
